# Emotion dysregulation and heart rate variability improve in US veterans undergoing treatment for posttraumatic stress disorder: Secondary exploratory analyses from a randomised controlled trial

**DOI:** 10.1186/s12888-022-03886-3

**Published:** 2022-04-15

**Authors:** Danielle C. Mathersul, Kamini Dixit, R. Jay Schulz-Heik, Timothy J. Avery, Jamie M. Zeitzer, Peter J. Bayley

**Affiliations:** 1 War Related Illness and Injury Study Center (WRIISC), Veterans Affairs Palo Alto Health Care System, Palo Alto, CA 94304 USA; 2 Department of Psychiatry and Behavioral Sciences, Stanford University School of Medicine, Stanford, CA 94305 USA; 3 Discipline of Psychology, Murdoch University, Building 440, 90 South Street, Murdoch, WA 6150 Australia; 4 Centre for Molecular Medicine and Innovative Therapeutics, Health Futures Institute, Murdoch University, Building 440, 90 South Street, Murdoch, WA 6150 Australia; 5 National Centre for Posttraumatic Stress Disorder (NCPTSD), Veterans Affairs Menlo Park Health Care System, Menlo Park, CA 94025 USA; 6 Mental Illness Research, Education and Clinical Center (MIRECC), Veterans Affairs Palo Alto Health Care System, Palo Alto, CA 94304 USA

**Keywords:** Posttraumatic stress disorder, PTSD, Emotion regulation, Heart rate variability, HRV, Yoga, Cognitive processing therapy

## Abstract

**Background:**

Emotion regulation (ER) is a key process underlying posttraumatic stress disorder (PTSD), yet, little is known about how ER changes with PTSD treatment. Understanding these effects may shed light on treatment processes.

**Methods:**

We recently completed a non-inferiority design randomised controlled trial demonstrating that a breathing-based yoga practice (Sudarshan kriya yoga; SKY) was not clinically inferior to cognitive processing therapy (CPT) across symptoms of PTSD, depression, or negative affect. Here, in secondary exploratory analyses (intent-to-treat *N* = 85; per protocol *N* = 59), we examined whether self-reported ER (Difficulties in Emotion Regulation Scale; DERS) and physiological ER (heart rate variability; HRV) improved with treatment for clinically significant PTSD symptoms among US Veterans.

**Results:**

DERS-Total and all six subscales improved with small-to-moderate effect sizes (*d* = .24–.66) following CPT or SKY, with no differences between treatment groups. Following SKY (but not CPT), HR max–min (average difference between maximum and minimum beats per minute), LF/HF (low-to-high frequency) ratio, and normalised HF-HRV (high frequency power) improved (moved towards a healthier profile; *d* = .42–.55).

**Conclusions:**

To our knowledge, this is the first study to demonstrate that a breathing-based yoga (SKY) improved *both* voluntary/intentional *and* automatic/physiological ER. In contrast, trauma-focused therapy (CPT) only reliably improved self-reported ER. Findings have implications for PTSD treatment and interventions for emotional disorders more broadly.

**Trial registration:**

Secondary analyses of ClinicalTrials.gov NCT02366403.

**Supplementary Information:**

The online version contains supplementary material available at 10.1186/s12888-022-03886-3.

## Background

Posttraumatic stress disorder (PTSD) is a debilitating mental health disorder that develops in some – but not all – individuals after exposure to a traumatic event [1]. Trauma-focused therapies – including cognitive processing therapy (CPT), prolonged exposure therapy (PE), imaginal exposure (IE), eye-movement desensitisation and reprocessing (EMDR), and trauma-focused cognitive behavioural therapy (TF-CBT) – are the recommended first-line, “gold standard”, evidence-based treatments for PTSD [2–7]. Yet, despite demonstrating significantly larger effect sizes than wait-list controls, psychopharmacological medications, or supportive therapy (average effect size = 0.43, n.s; [8]), up to two-thirds of individuals retain a PTSD diagnosis following trauma-focused therapy [9, 10].

Emotion regulation (ER) is a key process underlying many mental health disorders, including PTSD [11]. ER is a broad construct that encompasses awareness, comprehension (across cognitive, physiological, and behavioural manifestations), and adaptive and contextually appropriate responding to emotional experiences [11–14], which may be either voluntary/intentional or automatic [15]. Many of the symptoms of PTSD manifest as poor ER (often called “emotion dysregulation”; e.g., hyperarousal to threat, poor regulation of negative emotional states such as sadness or anger) [1] and a growing body of evidence highlights the role of ER in the development and maintenance of PTSD [16] and emotional disorders more broadly [11]. Indeed, a meta-analysis confirmed strong associations between symptoms of PTSD and self-report measures of difficulties in general ER – as well as specific sub-strategies of ER (rumination, thought suppression, and experiential avoidance) – independent of sample size or trauma type [17].

Yet, little is known about how ER changes with treatment for PTSD symptoms. Further, an over-reliance on self-report measures of ER exposes findings to reporting bias. Indeed, self-report measures may fail to identify ER deficits that exist in real-world settings [18, 19], especially among clinical populations where emotional insight and self-awareness may be reduced or lacking and/or in treatment studies where demand characteristics are particularly salient (i.e., participants may alter their self-reported experiences to appear a certain way post-intervention). Objective, biological methods increase ecological validity, inform mechanisms of pathology, and are an important adjunct to self-report measures. Heart rate variability (HRV) is a well-validated biomarker of ER capacity and flexibility in healthy adults [20, 21] and psychopathology more broadly [22] as well as PTSD specifically [23, 24]. Lower resting levels of HRV are proposed to reflect autonomic inflexibility and a vulnerability toward maladaptive responses to stressful or emotionally evocative experiences [25] (i.e., difficulties in ER). Indeed, studies of healthy adults demonstrate inverse relationships between self-reported difficulties in ER and both resting-state HRV [26] and ambulatory 24-h HRV [27]. A recent meta-analysis confirmed small but significant inverse associations between PTSD (self-report, clinical interview) and baseline HRV [28].

Here, we explored whether multi-modal ER improved with treatment for clinically significant PTSD symptoms among US Veterans. We present secondary exploratory analyses from a recently completed randomised controlled trial (RCT) showing that a breathing-based yoga practice (Sudarshan kriya yoga; SKY) was not clinically inferior to CPT at end-of-treatment for symptoms of PTSD, depression, and negative affect among US Veterans [29]. Self-reported ER (the Difficulties in Emotion Regulation Scale; DERS) and 5-min at-rest (sleep time) HRV (which we refer to here as “physiological ER”) were collected at baseline and end-of-treatment. Based on the primary outcomes study findings, we hypothesised that both self-reported and physiological ER would improve to a healthier profile with both CPT and SKY for PTSD (*Hypothesis A*). We also explored whether there were group treatment differences in multi-modal ER (*Hypothesis B*). On one hand, one might hypothesise greater changes in self-reported ER following CPT and greater changes in physiological ER following SKY, based on their specific targeted actions on intentional [11–14] and automatic regulation [30,31,32, 33], respectively. Alternatively, given extant literature demonstrating inverse relationships between self-reported difficulties in ER and HRV [26–28], one might hypothesise that regardless of whether the treatment targets conscious/intentional or automatic regulation, these treatment actions will manifest similarly in both self-reported and physiological ER across treatment groups.

## Methods

### Participants

Participants were US Veterans recruited from the San Francisco Bay Area via flyers and advertisements. All participants had clinically significant levels of PTSD symptoms (≥38 on the PTSD Checklist for DSM-5; PCL-5; [34]) and took part in the pre-registered non-inferiority RCT “Breathing Meditation Intervention for Post-Traumatic Stress Disorder” (ClinicalTrials.gov NCT02366403; *N* = 85 randomised; [29, 35]). Here, we report secondary exploratory intent-to-treat (ITT) and per protocol^1^ (≥ 75% treatment sessions) analyses on the self-reported and physiological ER data collected from the 85 randomised Veterans with PTSD (59 treatment completers; Table 1). Samples sizes were determined by power required to detect the non-inferiority threshold elsewhere [29, 35]. Approximately 10–25% of cardiac data were lost across time points due to poor data quality (see Results and Supplementary Figure 1). There were no significant differences between treatment groups in missing data or demographics (all *p* > .05) and no significant differences in DERS scores for those with or without cardiac data (all *p* > .29).

**Table 1:** Baseline demographics and clinical characteristics by treatment group for our treatment completers (per protocol; *N* = 59) sample

	CPT (*n* = 29)	SKY (*n* = 30)
Age	58.21 (13.04)	60.67 (11.00)
% Male	93.1	76.7
% White	65.5	63.3
% Married or Domestic Partner	41.4	36.7
% Bachelor’s degree or higher	27.6	23.3
CAPS-5 (total)	31.55 (14.24)	30.10 (12.45)
PCL-5 (total)	49.64 (9.00)	53.64 (11.58)

*CPT* Cognitive processing therapy, *SKY* Sudarshan kriya yoga, *CAPS-5* Clinician Administered PTSD Scale for DSM-5, *PCL-5* PTSD Checklist for DSM-5. Except where indicated by %, values are presented in the format *M (SD)*, where M = mean, SD = standard deviation. There were no significant differences between treatment groups on demographics or clinical characteristics at baseline. *The ITT sample (N = 85) demographics are presented in the primary outcomes manuscript* [29]

### Procedure

The protocol was approved by the Stanford University Institutional Review Board and conducted in accordance with the Declaration of Helsinki. The full procedure for the RCT is described elsewhere [29, 35]. Briefly, US Veterans with clinically significant levels of PTSD symptoms were randomised into either CPT or SKY groups and received their assigned intervention across a 6-week period. Per recommendations and protocols for each intervention, CPT was delivered via individual, twice-weekly sessions and SKY was delivered as an initial 5-day group workshop followed by ten, twice-weekly sessions across the remaining 5-weeks [29, 35]. Sessions were recorded and treatment adherence assessed. Participants were instructed to practice their respective intervention techniques on non-class days. All RCT participants were administered multiple clinician-administered, self-report, and physiological measures at multiple timepoints. Here, we report on self-reported ER (DERS) and physiological ER (HRV) collected at baseline and end-of-treatment.

### Measures

#### Self-reported ER: The Difficulties in Emotion Regulation Scale (DERS)

The DERS [14] is a 36-item self-report measure assessing clinically relevant emotional awareness, acceptance, comprehension, and adaptive and contextually appropriate responding to emotional experiences. Responses are rated on a 5-point Likert scale (0 = “almost never” to 5 = “almost always”), with higher scores reflecting poorer ER. The scale provides a measure of overall difficulties in ER (DERS-Total) as well as six subscales (as determined by the test constructors using factor analysis): DERS-Non-Acceptance (non-acceptance of emotional responses), DERS-Goals (difficulties engaging in goal-directed behaviour during negative emotional experiences), DERS-Impulse (impulse control difficulties in response to negative emotions), DERS-Awareness (lack of emotional awareness), DERS-Strategies (limited access to [effective] strategies), and DERS-Clarity (lack of emotional clarity). The DERS-Total and all six subscales demonstrate high internal consistency (Cronbach’s *α* = .80–.93) and good test-retest reliability (*ρ*_*I*_ = .57–.89).

#### Physiological ER: heart rate variability (HRV)

Continuous ambulatory cardiac data were collected over a 24-h period using Actiwave Cardio monitors (CamNtech Ltd), which are compact, lightweight, waterproof, chest-worn devices that record heart rate (bpm), inter-beat-interval (IBI), and physical activity. Data were pre-processed and extracted using Kubios HRV Premium 3.1.1 (Kubios, 2019) software for scientific research [37–40] that can calculate HRV from Actiwave data without the need to concurrently measure respiratory rate. Per standard recommendations, R-R intervals were also visually inspected for artefacts [41]. All data pre-processing was blind to treatment group.

Previous studies of continuous ambulatory cardiac data suggest night-time (sleep) periods have the greatest discriminatory power across different mental health disorders [42, 43] and between Veterans and non-Veterans [44, 45], likely because it represents a relatively behaviour-independent measure of general physiological ER capacity. We defined sleep timing using concurrently recorded triaxial accelerometer (actigraphy) data (Motionlogger, Ambulatory Monitoring, Ardsley NY) and validated algorithms [46] embedded in manufacturer-provided software (ActionW, Ambulatory Monitoring, Ardsley NY). We then extracted cardiac indices from a 5-min epoch of clean, artefact-free cardiac data during the first hour of sleep (likely non-rapid eye movement sleep). Per recommendations [41], we included multiple HRV indices across both time-domain (average difference between the maximum and minimum HR; [HR max–min (bpm)], square root of the mean squared differences between successive R-R intervals [RMSSD (ms)], standard deviation of the IBI of normal sinus beats [SDNN (ms)]) and frequency-domain (low-to-high frequency ratio [LF/HF], high frequency power [HF-HRV (normalised [FFT n.u.], absolute [FFT ms^2^])], low frequency peak [LF peak (Hz)], low frequency power [LF-HRV (absolute [FFT ms^2^]). Previous systematic reviews and meta-analyses have found significant associations between RMSSD, SDNN, and HF-HRV and PTSD [28, 47] and self-reported ER [20, 21].

HR max–min was used as an index of respiratory sinus arrythmia (RSA), where higher values reflect slower respiration rate [48]. Similarly, lower/higher values of LF peak/LF-HRV suggest a respiration rate approaching resonance frequency (slower HR oscillations), as typically observed following HRV biofeedback [48]. LF/HF ratio was used as an index of sympathetic/parasympathetic (sympathovagal) balance [49] representing vagally-mediated processes [48]. RMSSD is the most commonly used time-domain measure of HRV [48] and reflects general autonomic nervous system (ANS) function (predominantly parasympathetic activation; [41, 49]), with higher values typically associated with healthier function [49–52]. SDNN generally reflects parasympathetically-mediated RSA, is highly correlated with HR, and lower values predict morbidity and mortality [48]. We have previously found different patterns of results for normalised versus absolute HF-HRV [53] and note that studies do not always report on exact parameters. Thus, we include both HF-HRV values as indices of parasympathetic activation [44, 45], though normalised HF-HRV has also been proposed as a measure of ANS balance [41].

### Analyses

All analyses were conducted separately by ITT and per protocol, blind to treatment group, and conducted in IBM SPSS Statistics 27 with significance threshold set at *p* < .05. Cohen’s *d* and *β* estimates are reported as measures of effect size. Due to the exploratory nature of these secondary analyses and our a priori hypothesis-driven approach, we probed trend-level (*p* = .05–.10) interaction effects to highlight patterns that warrant further exploration and future replication (only effects sizes reported) and did not control for multiple comparisons. Simple correlations between change scores (baseline minus end-of-treatment) for physiological ER (HRV) and self-reported ER (DERS) are displayed in Supplementary Table 1.

We analysed separate repeated measures linear mixed models (LMM) for each of the self-reported and physiological ER measures. Time (baseline, end-of-treatment) was the repeated measures variable. Between-subjects variables were group (CPT, SKY; coded − 0.5, + 0.5 for ease of interpretation [54, 55]), self-reported ER (DERS Total and all subscales), and physiological ER (HR max–min, LF/HF, RMSSD, SDNN, normalised/absolute HF-HRV, LF peak, LF-HRV). Time was mean centred [54–57] and outliers (≥ ±3 SD) were Winsorized and replaced with the next highest value (× 2 for each of the HRV indices), per recommendations [58]. As all repeated measures had only two time points (baseline, end-of-treatment), we tested only the simplest covariance structure (compound symmetry [CS]; [56]).

All main and interactions effects were included in the models. Depending on the direction of change effects, a significant main effect of time would provide support for *Hypothesis A* (that both self-reported and physiological ER improved to a healthier profile with both CPT and SKY for PTSD), while a significant group by time interaction effect would provide support for *Hypothesis B* (i.e., group treatment differences in self-reported and/or physiological ER).

## Results

### Self-reported ER

**Fig. 1:**
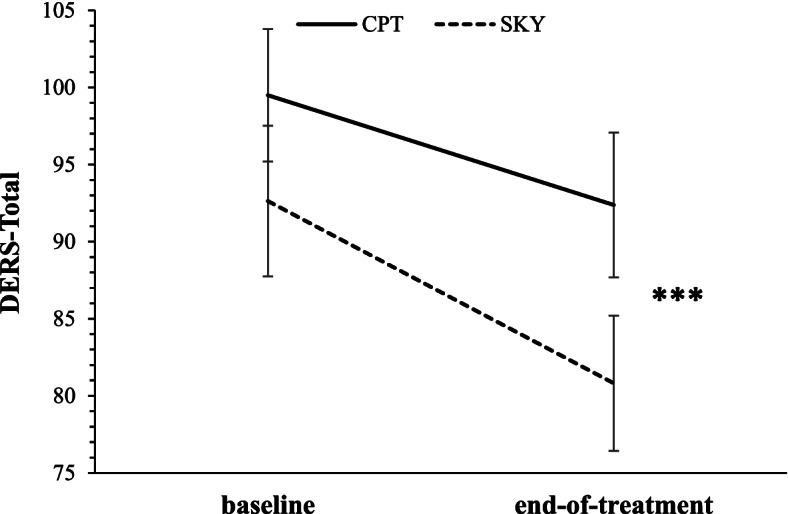
Mean total score on the Difficulties in Emotion Regulation Scale (DERS-Total) at baseline and end-of-treatment for Veterans who received either Sudarshan kriya yoga (SKY) or cognitive processing therapy (CPT) for PTSD (per protocol). Lower values reflect better emotion regulation. *** denotes significant (*p* < .001) main effect of time

**Fig. 2:**
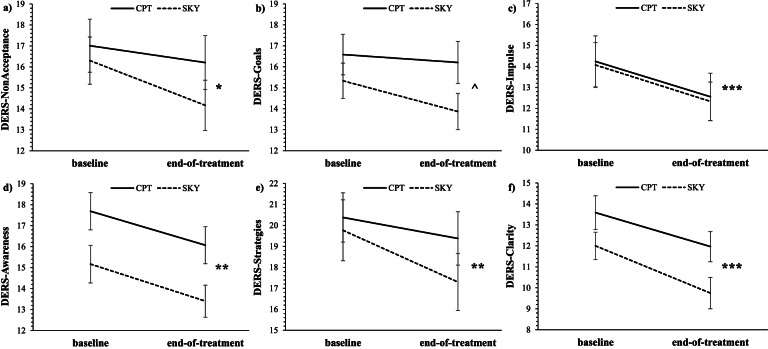
Mean self-reported scores for all six subscales of the Difficulties in Emotion Regulation Scale; **a**: non-acceptance of emotional responses (DERS-Non-Acceptance), **b**: difficulties engaging in goal-directed behaviour during negative emotional experiences (DERS-Goals), **c**: impulse control difficulties in response to negative emotions (DERS-Impulse), **d**: lack of emotional awareness (DERS-Awareness), **e**: limited access to effective strategies (DERS-Strategies), and **f**: lack of emotional clarity (DERS-Clarity) at baseline and end-of-treatment for Veterans who received either Sudarshan kriya yoga (SKY) or cognitive processing therapy (CPT) for PTSD (per protocol). For all DERS subscales, lower values reflect better emotion regulation. For main effects of time: *** *p* < .001, ** *p* < .01, * *p* < .05, ^ *p* = .05–.10

Overall, both CPT and SKY were associated with improvements in self-reported ER, with no differences between treatment groups. Findings were similar across both ITT and per protocol analyses (Table 2; per protocol analyses plotted in Figs. 1 and 2a-f; group means and standard deviations displayed in Supplementary Table 2).

**Table 2:** *Self-reported ER (DERS) effects for time and group by time for both ITT and per protocol analyses*

DERS domain	Time						Group x Time							
	*ITT (base n = 85; EOT n = 64)*			*Per Protocol (N = 59)*			*ITT (base n = 85; EOT n = 64)*				*Per Protocol (N = 59)*			
	*β*	*t*	*d*	*β*	*t*	*d*	*β*	*t*	CPT *d*	SKY *d*	*β*	*t*	CPT *d*	SKY *d*
Total	−1.32	−5.44***	.66	−1.18	−4.71***	.60	−.34	−.71	.55	.79	−.59	−1.17	.45	.76
Non-Acceptance	−.20	−2.77**	.34	−.18	−2.45*	.32	−.10	−.73	.20	.58	−.17	−1.10	.14	.61
Goals	−.15	−2.52*	.32	−.12	−1.89^	.24	−.10	−.82	.22	.41	−.14	−1.11	.10	.37
Impulse	−.25	−4.64***	.54	−.21	−4.12***	.53	.06	.52	.59	.48	−.01	−.05	.55	.51
Awareness	−.21	−3.25**	.39	−.21	−3.07**	.40	−.05	−.42	.35	.43	−.02	−.132	.40	.39
Strategies	−.27	−3.40**	.42	−.22	−2.78**	.36	−.09	−.59	.33	.53	−.18	−1.18	.21	.51
Clarity	−.24	−4.82***	.60	−.24	−4.41***	.57	−.05	−.47	.55	.63	−.08	−.72	.50	.63

*ER* Emotion regulation, *DERS* The Difficulties in Emotion Regulation Scale, *ITT* Intent-to-treat, *base* baseline, *EOT* End-of-treatment, *Non-Acceptance* Non-acceptance of emotional responses, *Goals* difficulties engaging in goal-directed behaviour during negative emotional experiences, *Impulse *impulse control difficulties in response to negative emotions, *Awareness* lack of emotional awareness, *Strategies* limited access to effective strategies, *Clarity* lack of emotional clarity, *CPT* Cognitive processing therapy, *SKY *Sudarshan kriya yoga. *** *p* < .001, ** *p* < .01, * *p* < .05, ^ *p* = .05–.10

#### DERS-Total

There was a significant main effect of time for Total DERS (ITT: *β* = − 1.32, *t* = − 5.44, *p* < .001; Cohen’s *d* = .66; per protocol: *β* = − 1.18, *t* = − 4.71, *p* < .001; Cohen’s *d* = .60) and no significant group by time interaction. In support of *Hypothesis A*, both treatment groups demonstrated significant improvements in self-reported ER by a moderate-to-large effect size (ITT: CPT Cohen’s *d* = .55; SKY Cohen’s *d* = .79; per protocol: CPT Cohen’s *d* = .45; SKY Cohen’s *d* = .76; Fig. 1; Table 2) and this improvement did not differ by treatment group (i.e., no support for *Hypothesis B*).

#### DERS-subscales

In support of *Hypothesis A*, both treatment groups, across both ITT and per protocol analyses, demonstrated improvements in self-reported ER across all DERS subscales ranging from small to moderate effect sizes. The largest effects were found for DERS-Impulse and DERS-Clarity (Fig. 2a-f; Table 2). There were no significant group by time interaction effects (i.e., no support for *Hypothesis B*).

### Physiological ER

**Fig. 3:**
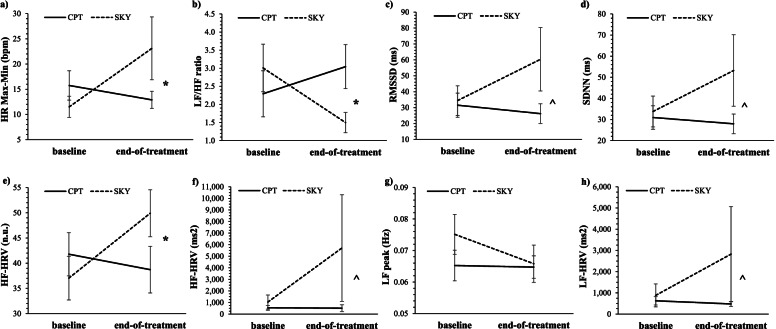
Mean physiological emotion regulation for all eight heart rate variability (HRV) indices at baseline and end-of-treatment for Veterans who received either cognitive processing therapy (CPT) or Sudarshan kriya yoga (SKY) for PTSD (per protocol). Top row: **a**: average difference between the maximum and minimum HR (HR max–min [bpm]), **b**: low-to-high frequency ratio (LF/HF), **c**: square root of the mean squared differences between successive R-R intervals (RMSSD [ms]), **d**: standard deviation of the IBI of normal sinus beats (SDNN [ms]). Bottom row: **e**: normalised high frequency power (HF-HRV [FFT n.u.]), **f**: absolute high frequency power (HF-HRV [FFT ms^2^]), **g**: peak frequency of the low frequency band (LF peak [Hz]), **h**: absolute low frequency power (LF-HRV [FFT ms^2^]). For all HRV indices except LF/HF ratio, higher values reflect better emotion regulation. * denotes significant (*p* < .05) group by time interaction effect. ^ denotes small-to-moderate effect size group difference at end-of-treatment

Overall, SKY was associated with greater treatment-related improvements in physiological ER than CPT, across both ITT and per protocol analyses, though only the per protocol analyses reached the significance threshold (Table 3) and were therefore plotted (Fig. 3a-h; group means and standard deviations displayed in Supplementary Table 3).

**Table 3:** Physiological ER (HRV) effects for time and group by time for both ITT and per protocol analyses

HRV index	Time						Group x Time							
	*ITT (base n = 63; EOT n = 54)*			*Per Protocol (base n = 45; EOT n = 49)*			*ITT (base n = 63; EOT n = 54)*				*Per Protocol (base n = 45; EOT n = 49)*			
	*β*	*t*	*d*	*β*	*t*	*d*	*β*	*t*	CPT *d*	SKY *d*	*β*	*t*	CPT *d*	SKY *d*
HR max-min	.33	.84	−.15	.57	1.32	−.28	1.21	1.54	.07	−.24	1.81	2.11*	.01	−.42
LF/HF	−.08	−1.48	.17	−.05	−.84	.17	−.18	−1.59	−.05	.49	−.25	−2.05*	−.04	.49
RMSSD	.89	.72	−.11	1.34	.97	−.18	2.77	1.13	.13	−.17	3.95	1.43	.14	−.28
SDNN	.52	.50	−.11	1.05	.90	−.17	2.17	1.05	.06	−.17	2.82	1.21	.06	−.26
HF-HRV (n.u.)	.74	1.76^	−.17	.64	1.34	−.22	1.37	1.62	.08	−.45	1.96	2.06*	.07	−.55
HF-HRV (ms^2^)	256.84	1.13	−.15	289.96	1.03	−.16	521.11	1.15	−.09	−.20	587.60	1.05	−.09	−.23
LF peak (Hz)	−.001	−1.03	.20	−.001	−1.06	.18	−.001	−.96	.02	.31	−.001	−.95	.03	.29
LF-HRV (ms^2^)	78.76	.70	−12	112.34	.82	−.13	255.80	1.14	.13	−.17	261.71	.95	.13	−.20

*ER* Emotion regulation, *ITT* Intent-to-treat, *base* Baseline, *EOT* End-of-treatment, *HRV* Heart rate variability; HR max-min = average difference between the maximum and minimum HR (bpm); LF/HF = low-to-high frequency ratio; RMSSD = square root of the mean squared differences between successive R-R intervals (ms); SDNN = standard deviation of the IBI of normal sinus beats (ms); HF-HRV (n.u.) = normalised high frequency power HRV (FFT); HF-HRV (ms^2^) = absolute high frequency power (FFT); LF peak (Hz) = peak frequency of the low frequency band (FFT); LF-HRV (ms^2^) = absolute low frequency power (FFT); CPT = cognitive processing therapy; SKY = Sudarshan kriya yoga. *** *p* < .001, ** *p* < .01, * *p* < .05, ^ *p* = .05–.10

#### HR max–min

There was no significant main effect of time for HR max–min (i.e., no support for *Hypothesis A*). There was a significant group by time interaction for HR max–min for the per protocol (*β* = 1.81, *t* = 2.11, *p* = .040) but not ITT analyses (*p* = .939). In support of *Hypothesis B*, the SKY group, by a moderate effect size (Cohen’s *d* = −.42), demonstrated a larger increase than the CPT group (Cohen’s *d* = .01) from baseline to end-of-treatment and larger overall at end-of-treatment (ITT: Cohen’s d = −.46; per protocol: Cohen’s d = −.47) in the average difference between minimum and maximum HR (per protocol; Fig. 3a; Table 3).

#### LF/HF ratio

There was no significant main effect of time for LF/HF (i.e., no support for *Hypothesis A*). There was a significant group by time interaction for LF/HF for the per protocol (*β* = −.25, *t* = − 2.05, *p* = .049) but not ITT analyses (*p* = .120), though follow-up effect sizes were similar for both analyses. In support of *Hypothesis B*, the SKY group, by a moderate effect size (ITT: Cohen’s *d* = .49; per protocol: Cohen’s *d* = .49), had greater reductions in LF/HF ratio from baseline to end-of-treatment (ITT: Cohen’s *d* = −.05; per protocol: Cohen’s *d* = −.04) and had lower LF/HF ratio (i.e., better physiological ER) at end-of-treatment (ITT: Cohen’s *d* = .53; per protocol: Cohen’s *d* = .63) than the CPT group (Fig. 3b; Table 3).

#### RMSSD/SDNN

There were no significant effects for RMSSD or SDNN for either ITT or per protocol analyses. Follow-up exploratory tests suggested the SKY group, by a moderate effect size, tended to have higher RMSSD (ITT: Cohen’s *d* = −.50; per protocol: Cohen’s *d* = −.52; Fig. 3c) and SDNN (ITT: Cohen’s *d* = −.44; per protocol: Cohen’s *d* = −.47; Fig. 3d) at end-of-treatment (i.e., better physiological ER) than the CPT group (Table 3; *Hypothesis B*), despite no differences at baseline.

#### HF-HRV

There were no significant main effects of time for normalised or absolute HF-HRV (i.e., no support for *Hypothesis A*). There was a significant group by time interaction for normalised HF-HRV for the per protocol (*β* = 1.96, *t* = 2.06, *p* = .045) but not ITT analyses (*p* = .110), though follow-up effect sizes were similar for both analyses. In support of *Hypothesis B*, the SKY group, by a moderate effect size (ITT: Cohen’s *d* = −.45; per protocol: Cohen’s *d* = −.55), had larger increases in normalised HF-HRV from baseline to end-of-treatment (ITT: Cohen’s *d* = .08; per protocol: Cohen’s *d* = .07) and had higher normalised HF-HRV (i.e., better physiological ER) at end-of-treatment (group (ITT: Cohen’s *d* = −.47; per protocol: Cohen’s *d* = −.49) than the CPT group (Fig. 3e; Table 3). Follow-up exploratory tests suggested the SKY group, by a small-to-moderate effect size (ITT: Cohen’s *d* = −.37; per protocol: Cohen’s *d* = −.38), also had higher absolute HF-HRV (i.e., better physiological ER) at end-of-treatment than the CPT group (Fig. 3f; Table 3; *Hypothesis B*), despite no differences at baseline.

#### LF band

There were no significant effects for LF peak or LF-HRV for either ITT or per protocol analyses. Follow-up exploratory tests suggested the SKY group, by a small-to-moderate effect size, had greater reductions in LF peak from baseline to end-of-treatment (ITT: Cohen’s d = .31; per protocol: Cohen’s d = .29; Fig. 3g) and had higher LF-HRV at end-of-treatment (i.e., better physiological ER; ITT: Cohen’s *d* = −.31; per protocol: Cohen’s *d* = −.32) than the CPT group (Fig. 3h; Table 3; *Hypothesis B*), despite no differences at baseline.

## Discussion

To our knowledge, this is the first study to investigate the effects of PTSD treatment on *both* self-reported ER *and* physiological ER. We found that self-reported ER (measured by the DERS total score and all six subscales) improved following either SKY or CPT for PTSD, with the strongest effects for overall (total) difficulties in ER, followed by emotional clarity and impulse control. In contrast, physiological ER (5-min at-rest [sleep time] HRV) demonstrated modest improvements (i.e., moved towards a healthier profile) following SKY but not CPT for PTSD. Significant improvements were found for HR max–min, LF/HF ratio, and normalised HF-HRV for per protocol analyses; the remaining physiological ER indices (RMSSD, SDNN, absolute HF-HRV, LF peak, LF-HRV) showed numerical improvement across both ITT and per protocol but did not reach statistical significance.

Our primary outcomes manuscript showed that a breathing-based yoga (SKY) was not clinically inferior to a first-line PTSD treatment (CPT) for symptoms of PTSD, depression, and negative affect among US Veterans [29], using both ITT and per protocol analyses. Here, we demonstrate in secondary exploratory analyses (ITT *N* = 85; per protocol *N* = 59) that self-reported ER improved following SKY and CPT treatment (*Hypothesis A*). Improvements in self-reported ER alongside primary (PTSD) and comorbid (depression, negative affect) symptom outcomes are consistent with literature suggesting ER is a key treatment outcome that improves even when treatments do not directly target emotional processing [12, 59]. That self-reported ER improves with CPT – a TF-CBT – is perhaps unsurprising when CBT broadly (and CPT specifically) focuses on increasing adaptive ER strategies like cognitive reappraisal [12, 60, 61]. Perhaps more surprisingly, we found that self-reported ER also improved following treatment with SKY, a style of yoga predominantly consisting of regulated, cyclical breathing meditation exercises. Yoga is a holistic practice combining meditation (*dhyana*), focused non-judgmental attention (*dharana*), regulated breathing (*pranayama*), and physical postures (*asanas*). Some yoga practices like mindfulness (*dhyana/dharana* collectively) are widely utilised as sole, free-standing exercises or combined with psychological interventions like CBT. It could be argued that cultivating non-judgmental awareness and non-reactivity should improve ER, and indeed, some systematic reviews conclude mindfulness reduces neural emotional reactivity [30, 62, 63]. Yet, many yoga/mindfulness studies are limited by poor methodological design, including small sample size, inadequate/absent control/comparison, or cross-sectional non-clinical samples. Furthermore, for those studies demonstrating self-reported improvements in positive/negative affect and stress reactivity with the so-called “third-wave” mindfulness-based psychological interventions [64, 65], one cannot dismantle the unique effects of mindfulness versus CBT on ER. One review of yoga for ER found reduced emotional reactivity and increased use of adaptive coping strategies [66] and another found increased positive affect and improved mental health symptoms in healthy controls and individuals with physical health conditions [67]. Yet no study has explored yoga for ER in populations with clinically significant mental health symptoms. Thus, to our knowledge, this is the first study to demonstrate that a breathing-based yoga (SKY) improved self-reported difficulties in overall ER – as well as emotional clarity and impulse control – among individuals with clinically significant PTSD symptoms.

We explored whether there were group treatment differences in multi-modal ER (*Hypothesis B*) and found some support that SKY had stronger impact on improving physiological ER than CPT. Specifically, for our per protocol analyses, SKY significantly increased HR max–min and normalised HF-HRV and significantly reduced LF/HF ratio (alongside trend-level/moderate effect size improvements in RMSSD, SDNN, absolute HF-HRV, LF peak, LF-HRV). This adds to a growing body of literature suggesting yoga (including breathing-based meditation and mindfulness-based interventions) can improve ANS regulation and balance [30,31,32,67, 68]. This is also consistent with the HRV biofeedback literature that demonstrates slow, deep, resonance breathing of around 10s per breath increases HRV [69], improves emotion regulation [70], and reduces clinical symptoms of PTSD [71]. To our knowledge, this is the first study to demonstrate that SKY improves *both* voluntary/intentional (self-reported) *and* automatic (physiological) ER in individuals with clinically significant PTSD symptoms. Interestingly, normalised HF-HRV showed more robust findings than absolute HF-HRV, consistent with the notion that it is a more valid measure than absolute HF-HRV for between-subject comparisons (like treatment group by time interactions) [48]. Indeed, we previously found different patterns of findings for absolute versus normalised HF-HRV [53] and note that studies do not always report on exact parameters – nor do they report multiple time- and frequency-domain indices – highlighting the need for consistency, breadth, and replication across HRV indices. While our ITT analyses did not reach significance threshold for any HRV indices, exploratory follow-up effect size comparisons demonstrated similar patterns to the per protocol analyses, particularly for SKY versus CPT, with the strongest effects for per protocol. Consistent with our primary outcomes study that found larger overall effects for completers compared to the ITT analyses [29], our findings here suggest that completing the full SKY treatment protocol is necessary to achieve the strongest impact on autonomic regulation.

CPT did not appear to improve physiological ER in our study, despite significant improvements in self-reported ER. This apparent lack of coherence between self-reported and physiological ER is not uncommon (e.g., [72]) and is further reflected in the change score correlations between self-reported and physiological ER (Supplementary Table 1) which were largely non-significant and in the opposite direction than expected for CPT (i.e., improvements in self-reported ER were associated with poorer physiological ER). Extant literature hints that CBT for PTSD may influence ANS function/physiological ER, though findings are mixed. For example, a meta-analysis of three studies found CBT for PTSD significantly reduced HR compared to treatment as usual or wait-list control [73]. In a more recent systematic review, nine out of 17 studies (seven TF-CBT) found treatment-related reductions in resting HR/HR reactivity while the remaining eight out of 17 (four TF-CBT) failed to demonstrate treatment-related changes in HR [74]. While this systematic review also found treatment-related increases in resting HRV in five out of six studies, only one of these [75] utilised a TF-CBT (combined CPT plus CBT for substance use disorder) and we could only see that this study examined baseline HRV as a predictor – not outcome – of treatment. Our study found no change in HRV indices following CPT. It is possible that the number of contact hours contributed to group treatment differences in physiological ER as SKY involves more total hours than CPT. However, both interventions were delivered in their most effective, standardised format, with CPT as an individual one-on-one therapy [76] and SKY as a group, so one could argue that CPT was more “concentrated” in our RCT [35]. Thus, while it appears that CPT does not improve physiological ER, further studies are needed.

Veterans achieved clinically meaningful reductions in PTSD symptoms with either CPT or SKY in our primary outcomes RCT [29]. Here, we demonstrated statistically significant improvements in self-reported and physiological ER via secondary exploratory analyses. Yet the question remains whether these treatment-related changes were *clinically meaningful*. One RCT set the DERS-Total cut-off at 96, one standard deviation (19.52) above the pooled grand mean (77.33) across several clinical and non-clinical studies published prior to July 2010 [77]. Our CPT and SKY Veterans were, on average, just above or just below this clinical cut-off, respectively, at baseline, and both groups were below at end-of-treatment. Our effect sizes for self-reported ER were small to moderately large (*d* = .24–.66) across all sub-scales and both ITT and per protocol analyses, within the range found in a systematic review and meta-analysis (*d* = 0.18–2.87), where the highest effect sizes were for treatments specifically targeted at improving ER [12]. Together, this suggests both SKY and CPT produced clinically meaningful improvements in self-reported ER. Regarding HRV norms [78], for RMSSD, both treatment groups began within the healthy range for 50–59-year-olds; after 6 weeks of treatment, the CPT group were still within this range while the SKY group were even higher (better/healthier) than their age-matched norms, closer to the norms of adults at least 10 years younger. It is possible that the lack of significant effects for RMSSD – especially for the CPT group – were driven by clinical ceiling effects limiting capacity for change. In contrast, for SDNN, both groups were significantly lower (i.e., less healthy) than the norms [78], both before and after treatment. Although we could not find studies reporting HF-HRV or LF/HF norms, mean treatment-related improvements for SKY were at least as large as average reported differences between PTSD and healthy profiles [24]. These gaps highlight the need for further research to establish clinically meaningful HRV thresholds.

Collectively, our findings support continued use of self-report measures of ER in clinical research (given the consistency across DERS domains, a total measure is likely sufficient). More ecologically valid measures such as ecological momentary assessment (EMA; also called experience sampling method, ESM) are recommended alongside these self-report questionnaires. Further validation of HRV indices alongside other measures of ER and clinical symptomology are also warranted to increase precision. For example, future studies might explore relationships between different PTSD symptom clusters (e.g., negative alterations in cognition and mood, altered ANS arousal/reactivity) and different types and measures of ER (e.g., self-report questionnaires, EMA/ESM, various HRV indices, reactivity [experimental, behavioural, neurophysiological]) and how these change with treatment (e.g., moderation/mediation analyses).

The major strength of this study is the analysis of changes in both self-reported and physiological ER with treatment for PTSD symptoms. Our findings are consistent with a growing body of literature supporting ER as a key treatment outcome across emotional disorders including PTSD [12, 59], regardless of treatment type or specific treatment target. Ambulatory physiological measures are more ecologically valid than laboratory-based assessments and it is noteworthy that our measures were collected during sleep, ruling out the conscious engagement of breathing techniques that may have occurred if we had collected these measures during wakefulness. A further strength is our use of both per protocol (treatment completer) and ITT analyses, per RCT recommendations [36]. Finally, we analysed multiple HRV indices across time- and frequency-domain, per recommendations [41], rather than select only those that supported our hypotheses.

The major limitation of this study is the large amount of missing HRV data across timepoints, which may account for the smaller number of significant effects for physiological ER. While missing data are common when using ambulatory methods of data collection, future studies should consider power issues when weighing up such advantages and disadvantages. Further, as we used an ambulatory device to record HRV, we did not have a concurrent measure of respiration. Although Kubios HRV software has well-established algorithms that can calculate HRV from Actiwave data without the need to concurrently measure respiratory rate [37–40], there remains debate in the field surrounding the need to further control for respiration when measuring HRV. Future replication studies could employ multiple different methods for comparison. Finally, it is important to note that while HRV is considered a well-validated biomarker of ER capacity and flexibility [20–25] and demonstrates inverse relationships with self-reported difficulties in ER [26–28] it remains relatively non-specific. Indeed, lower HRV is associated not only with difficulties in ER and poor mental health but cognitive deficits and poor physical health more broadly [49–52, 79]. Thus, while we have used HRV here as a proxy for physiological ER, an alternative proposal could be as a general biomarker of wellness.

## Conclusions

To our knowledge, this is the first study to demonstrate that a breathing-based yoga practice (SKY) for PTSD improves *both* voluntary/intentional (self-reported overall difficulties and specific difficulties with emotional clarity and impulse control) *and* automatic/physiological ER (strongest effects for HR max–min, normalised HF-HRV, and LF/HF ratio). Trauma-focused therapy (i.e., CPT) only reliably improved self-reported ER. As ER is a key process underlying many mental health disorders – not just PTSD – these findings have implications for emotional disorder treatment more broadly.

## Supplementary Information

**Additional file 1.** Supplementary Tables.

**Additional file 2.** Supplementary Figure.

## Data Availability

The datasets generated and/or analysed during the current study are not publicly available due to institutional regulations protecting service member data but are available from the corresponding author on reasonable written request (may require data use agreements to be developed).

## Abbreviations

ANS: Autonomic nervous system
CBT: Cognitive behavioural therapy
CPT: Cognitive processing therapy
DERS: Difficulties in Emotion Regulation Scale
ER: Emotion regulation
HF-HRV: High frequency power heart rate variability
HR max–min: Average difference between the maximum and minimum heart rate
HRV: Heart rate variability
IBI: Inter-beat-interval of heart rate
ITT: Intent-to-treat
LF/HF: Low-to-high frequency ratio heart rate variability
LF peak: Peak frequency for the low frequency band
LF-HRV: Low frequency power heart rate variability
PTSD: Posttraumatic stress disorder
RCT: Randomised controlled trial
RSA: Respiratory sinus arrythmia
RMSSD: Square root of the mean squared differences between successive R-R intervals
SDNN: Standard deviation of the inter-beat-interval of normal sinus beats
SKY: Sudarshan kriya yoga

## Footnotes

1 The primary outcomes RCT was a noninferiority design [29, 35], where using both a per protocol (treatment completer) analysis and an ITT analysis is recommended as the most rigorous approach to test primary outcomes [36].
