# Supplementary material for: Emotion dysregulation and heart rate variability improve in US veterans undergoing treatment for posttraumatic stress disorder: Secondary exploratory analyses from a randomised controlled trial
Source: BMC Psychiatry. 2022 Apr 15;22:268. doi: 10.1186/s12888-022-03886-3 (PMC9012004; doi:10.1186/s12888-022-03886-3)
Supplement: Supplementary file 1 — Additional file 1. Supplementary Tables. [file 12888_2022_3886_MOESM1_ESM.docx]

Supplementary Table 1

*Correlations between change scores (baseline minus end-of-treatment) for physiological ER (HRV) and self-reported ER (DERS)*

| DERS domain & HRV index | CPT | | | | | | |  | SKY | | | | | | |
| --- | --- | --- | --- | --- | --- | --- | --- | --- | --- | --- | --- | --- | --- | --- | --- |
|  | Total | Non-Acceptance | Goals | Impulse | Awareness | Strategies | Clarity |  | Total | Non-Acceptance | Goals | Impulse | Awareness | Strategies | Clarity |
| HR max-min | -0.10 | 0.03 | 0.02 | 0.13 | -0.19 | 0.02 | -0.47* |  | -0.11 | -0.27 | -0.20 | 0.08 | -0.20 | 0.10 | 0.05 |
| LF/HF | -0.67** | -0.59* | -0.43^ | -0.16 | -0.58* | -0.27 | -0.42^ |  | 0.36 | -0.18 | 0.22 | 0.27 | 0.51* | 0.29 | 0.23 |
| RMSSD | 0.06 | -0.01 | -0.02 | -0.18 | 0.14 | 0.16 | 0.11 |  | -0.18 | -0.21 | -0.02 | -0.03 | -0.21 | -0.19 | -0.06 |
| SDNN | 0.08 | 0.01 | -0.05 | -0.10 | 0.15 | 0.16 | 0.10 |  | -0.12 | -0.21 | 0.01 | 0.01 | -0.20 | -0.09 | 0.02 |
| HF-HRV (n.u.) | 0.28 | 0.14 | 0.03 | 0.07 | 0.44^ | -0.03 | 0.46^ |  | -0.39 | -0.02 | -0.17 | -0.32 | -0.38 | -0.44^ | -0.17 |
| HF-HRV (ms²) | -0.01 | -0.17 | -0.22 | -0.37 | 0.25 | 0.16 | 0.31 |  | -0.06 | -0.20 | 0.06 | -0.05 | -0.16 | 0.00 | 0.13 |
| LF peak (Hz) | -0.13 | -0.04 | -0.16 | -0.08 | 0.19 | -0.34 | -0.09 |  | 0.06 | -0.20 | 0.15 | 0.16 | 0.17 | -0.03 | -0.05 |
| LF-HRV (ms²) | 0.09 | 0.01 | 0.06 | -0.12 | 0.15 | 0.16 | 0.07 |  | -0.02 | -0.19 | 0.08 | -0.01 | -0.14 | 0.05 | 0.16 |

*Note*. ER = emotion regulation; HRV = heart rate variability; DERS = the Difficulties in Emotion Regulation Scale; CPT = cognitive processing therapy; SKY = Sudarshan kriya yoga; Non-Acceptance = non-acceptance of emotional responses; Goals = difficulties engaging in goal-directed behaviour during negative emotional experiences; Impulse = impulse control difficulties in response to negative emotions; Awareness = lack of emotional awareness; Strategies = limited access to effective strategies; Clarity = lack of emotional clarity; HR max-min = average difference between the maximum and minimum HR (bpm); LF/HF = low-to-high frequency ratio; RMSSD = square root of the mean squared differences between successive R-R intervals (ms); SDNN = standard deviation of the IBI of normal sinus beats (ms); HF-HFV (n.u.) = normalised high frequency power HRV (FFT); HF-HFV (ms²) = absolute high frequency (FFT); LF peak (Hz) = peak frequency of the low frequency band (FFT); LF-HFV (ms²) = absolute low frequency power (FFT). *For all HRV indices except LF/HF ratio, negative correlations indicate concordance between physiological and self-reported ER in that better emotion regulation on an HRV index tracks with better emotion regulation on a DERS domain*. *** *p* < .001, ** *p* < .01, * *p* < .05, ^ *p* = .05-.10.

Supplementary Table 2

*Self-reported ER (DERS) group averages at baseline and EOT for both ITT and per protocol analyses*

| DERS domain | CPT | | | |  | SKY | | | |
| --- | --- | --- | --- | --- | --- | --- | --- | --- | --- |
|  | *ITT* | | *Per Protocol* | |  | *ITT* | | *Per Protocol* | |
|  | *Base (n=44)* | *EOT (n=32)* | *Base (N=29)* | *EOT (N=29)* |  | *Base (n=41)* | *EOT (n=32)* | *Base (N=30)* | *EOT (N=30)* |
| Total | 101.80 (23.86) | 92.91 (24.58) | 99.50 (23.14) | 92.38 (25.30) |  | 95.59 (27.04) | 83.42 (25.67) | 92.63 (26.76) | 80.82 (24.03) |
| Non-Acceptance | 17.53 (6.51) | 16.34 (6.75) | 17.01 (6.82) | 16.21 (6.91) |  | 16.49 (6.51) | 14.59 (6.80) | 16.30 (6.17) | 14.17 (6.56) |
| Goals | 16.80 (4.91) | 16.13 (5.17) | 16.59 (5.22) | 16.21 (5.39) |  | 15.66 (4.78) | 14.19 (4.76) | 15.33 (4.63) | 13.87 (4.75) |
| Impulse | 14.93 (6.31) | 12.66 (5.87) | 14.24 (6.52) | 12.55 (6.07) |  | 15.34 (6.29) | 12.81 (5.33) | 14.07 (5.86) | 12.33 (5.04) |
| Awareness | 17.91 (4.70) | 16.44 (4.71) | 17.69 (4.78) | 16.07 (4.75) |  | 15.56 (5.24) | 13.66 (4.17) | 15.17 (4.91) | 13.40 (4.18) |
| Strategies | 20.82 (6.89) | 19.28 (6.59) | 20.38 (6.33) | 19.38 (6.86) |  | 20.46 (8.00) | 18.03 (7.82) | 19.77 (7.96) | 17.30 (7.45) |
| Clarity | 13.82 (4.33) | 12.06 (3.73) | 13.59 (4.33) | 11.97 (3.88) |  | 12.07 (3.50) | 10.14 (4.26) | 12.00 (3.56) | 9.75 (4.10) |

*Note*. ER = emotion regulation; DERS = the Difficulties in Emotion Regulation Scale; ITT = intent-to-treat; base = baseline; EOT = end-of-treatment; Non-Acceptance = non-acceptance of emotional responses; Goals = difficulties engaging in goal-directed behaviour during negative emotional experiences; Impulse = impulse control difficulties in response to negative emotions; Awareness = lack of emotional awareness; Strategies = limited access to effective strategies; Clarity = lack of emotional clarity; CPT = cognitive processing therapy; SKY = Sudarshan kriya yoga. *Data are in the form of* *means (standard deviations).*

Supplementary Table 3

*Physiological ER (HRV) group averages at baseline and EOT for both ITT and per protocol analyses*

| HRV index | CPT | | | |  | SKY | | | |
| --- | --- | --- | --- | --- | --- | --- | --- | --- | --- |
|  | *ITT* | | *Per Protocol* | |  | *ITT* | | *Per Protocol* | |
|  | *Base (n=33)* | *EOT (n=29)* | *Base (N=21)* | *EOT (N=26)* |  | *Base (N=30)* | *EOT (N=25)* | *Base (N=24)* | *EOT (N=23)* |
| HR max-min | 14.99 (11.54) | 12.80 (8.60) | 15.75 (13.32) | 12.90 (8.73) |  | 14.81 (15.69) | 22.27 (28.78) | 11.51 (10.30) | 23.11 (29.91) |
| LF/HF | 3.15 (3.42) | 3.11 (3.01) | 2.29 (2.92) | 3.04 (3.11) |  | 3.31 (3.57) | 1.76 (1.84) | 3.01 (3.21) | 1.50 (1.34) |
| RMSSD | 29.76 (33.67) | 25.72 (30.19) | 31.52 (34.78) | 26.20 (31.84) |  | 38.78 (51.22) | 56.91 (92.07) | 34.41 (45.57) | 60.37 (95.34) |
| SDNN | 32.58 (30.09) | 27.97 (22.69) | 30.95 (25.49) | 27.95 (23.72) |  | 38.13 (39.08) | 50.91 (78.14) | 33.81 (35.73) | 53.22 (81.18) |
| HF-HRV (n.u.) | 35.84 (19.56) | 37.24 (22.90) | 41.79 (19.65) | 38.72 (23.62) |  | 36.27 (21.09) | 47.96 (22.78) | 37.03 (21.18) | 49.90 (22.30) |
| HF-HRV (ms²) | 506.57 (1026.40) | 476.83 (1420.44) | 539.25 (983.02) | 508.55 (1498.77) |  | 1123.49 (2916.83) | 5262.66 (21241.31) | 1039.50 (2978.58) | 5709.58 (22126.92) |
| LF peak (Hz) | 0.06 (0.02) | 0.06 (0.02) | 0.07 (0.02) | 0.06 (0.02) |  | 0.07 (0.03) | 0.07 (0.03) | 0.08 (0.03) | 0.07 (0.03) |
| LF-HRV (ms²) | 872.09 (1592.78) | 479.01 (587.43) | 625.64 (894.91) | 477.49 (612.65) |  | 982.30 (1685.24) | 2635.60 (10312.79) | 879.49 (1759.96) | 2824.99 (10749.38) |

*Note*. ER = emotion regulation; HRV = heart rate variability; ITT = intent-to-treat; base = baseline; EOT = end-of-treatment; HR max-min = average difference between the maximum and minimum HR (bpm); LF/HF = low-to-high frequency ratio; RMSSD = square root of the mean squared differences between successive R-R intervals (ms); SDNN = standard deviation of the IBI of normal sinus beats (ms); HF-HFV (n.u.) = normalised high frequency power HRV (FFT); HF-HFV (ms²) = absolute high frequency power (FFT); LF peak (Hz) = peak frequency of the low frequency band (FFT); LF-HFV (ms²) = absolute low frequency power (FFT); CPT = cognitive processing therapy; SKY = Sudarshan kriya yoga. *Data are in the form of* *means (standard deviations).*
