# Supplementary material for: Emotion dysregulation and heart rate variability improve in US veterans undergoing treatment for posttraumatic stress disorder: Secondary exploratory analyses from a randomised controlled trial
Source: BMC Psychiatry. 2022 Apr 15;22:268. doi: 10.1186/s12888-022-03886-3 (PMC9012004; doi:10.1186/s12888-022-03886-3)
Supplement: Supplementary file 2 — Additional file 2. Supplementary Figure. [file 12888_2022_3886_MOESM2_ESM.docx]

**SKY**

**CPT**

**Enrollment**

Excluded (n = 135)

- Declined (n = 82)
- Did not meet eligibility criteria (n = 53)

Randomised (*N* = 92)

ITT (*N* = 85)

- Removed by investigator (n = 7)
  - 5 ineligible
  - 2 cancelled due to low enrolment

Completed PCL-5 Ph Screen (n = 227)

Assessed for eligibility via on site screen (n = 144)

**Baseline**

**End-of-Treatment**

**Data Analysis**

- **ITT**
  - 41 DERS data
  - 30 HRV data
- **Per protocol**
  - 30 DERS data
  - 25 HRV data
- **32 Completed Assessment**
  - 30 “treatment completers”
  - 32 DERS data
  - 25 HRV data (7 missing)

**41 Allocated to SKY**

- - 41 DERS data
  - 30 HRV data (11 missing)
- **32 Completed Assessment**
  - 29 “treatment completers”
  - 32 DERS data
  - 29 HRV data (3 missing)
- **44 Allocated to CPT**
  - 44 DERS data
  - 33 HRV data (11 missing)
- **ITT**
  - 44 DERS data
  - 33 HRV data
- **Per protocol**
  - 29 DERS data
  - 29 HRV data

Supplementary Figure 1. Consort diagram. *Note*. ITT = intent to treat; CPT = cognitive processing therapy; SKY = Sudarshan kriya yoga; DERS = the Difficulties in Emotion Regulation Scale; HRV = heart rate variability. Bolded, first-level bullet-points indicate total number of participants who completed assessments at each stage. Second-level bullet-points indicate additional reasons for lost data (e.g., equipment failure [missing] or poor data quality). Note that with an intent-to-treat design, we aimed to obtain assessments from all randomised participants at all treatment time points.
